# A longitudinal twin study of the association between childhood autistic traits and psychotic experiences in adolescence

**DOI:** 10.1186/s13229-015-0037-9

**Published:** 2015-07-22

**Authors:** Mark J. Taylor, Elise B. Robinson, Francesca Happé, Patrick Bolton, Daniel Freeman, Angelica Ronald

**Affiliations:** Centre for Brain and Cognitive Development, Department of Psychological Sciences, Birkbeck, University of London, London, UK; Analytic and Translational Genetics Unit, Department of Medicine, Massachusetts General Hospital and Harvard Medical School, Boston, MA USA; Stanley Center for Psychiatric Research and Program in Medical and Population Genetics, Broad Institute of Harvard and MIT, Cambridge, MA USA; MRC Social, Genetic and Developmental Psychiatry Centre, Institute of Psychiatry, Psychology & Neuroscience, King’s College, London, UK; Department of Child Psychiatry and MRC SGDP Centre, Institute of Psychiatry, Psychology & Neuroscience, King’s College, London, UK; Department of Psychiatry, University of Oxford, Oxford, UK

**Keywords:** Autism, Psychotic experiences, Twin study, Genetics, Comorbidity

## Abstract

**Background:**

This twin study investigated whether autistic traits during childhood were associated with adolescent psychotic experiences.

**Methods:**

Data were collected from a community sample of approximately 5000 twin pairs, which included 32 individuals with diagnosed autism spectrum conditions (ASC). Parents rated autistic traits in the twins at four points between ages 8–16 years. Positive, negative, and cognitive psychotic experiences were assessed at age 16 years using self- and parent-report scales. Longitudinal twin analyses tested the associations between these measures.

**Results:**

Autistic traits correlated weakly or nonsignificantly with positive psychotic experiences (paranoia, hallucinations, and grandiosity), and modestly with cognitive psychotic experiences (cognitive disorganisation). Higher correlations were observed for parent-rated negative symptoms and self-reported anhedonia, although the proportion of variance in both accounted for by autistic traits was low (10 and 31 %, respectively). The majority of the genetic influences on negative symptoms and anhedonia were independent of autistic traits. Additionally, individuals with ASC displayed significantly more negative symptoms, anhedonia, and cognitive disorganisation than controls.

**Conclusions:**

Autistic traits do not appear to be strongly associated with psychotic experiences in adolescence; associations were also largely restricted to negative symptoms. Of note, the degree to which the genetic and environmental causes of autistic traits influenced psychotic experiences was limited. These findings thus support a phenotypic and etiological distinction between autistic traits and psychotic experiences.

**Electronic supplementary material:**

The online version of this article (doi:10.1186/s13229-015-0037-9) contains supplementary material, which is available to authorized users.

## Background

Autism spectrum conditions (ASC) are characterised by atypical social communication and behavioural inflexibility [1]. While historically viewed as childhood schizophrenia, evidence on psychotic disorders in individuals with ASC is mixed. While some studies report a very low prevalence of psychotic disorders amongst individuals with ASC [2–4], others have reported prevalence as high as 13 % [5].

Psychotic experiences are milder manifestations of psychotic symptoms that commonly present in adolescence [6–9]. While evidence on clinical psychotic disorders in individuals with ASC is inconsistent, psychotic experiences appear relatively commonly in individuals with ASC [10, 11]. In two studies, negative symptoms were elevated in young children with ASC [12, 13]. In another, 5.4 % of children with ASC reported hallucinations [14]. Thus, the findings to date suggest that particular psychotic experiences may present relatively frequently in children with ASC.

Psychotic experiences are associated with elevated distress, suicide risk, and subsequent psychotic disorders [15–17], and so, there is a need to understand why they co-occur with ASC. There is some indication of overlapping causes between clinical psychotic disorders and ASC. ASC and schizophrenia seem to run together in the same families [18], and there is tentative evidence that certain genes may link with both conditions [19]. The complete picture is not clear, however; certain copy-number variants are unique to each condition, while a low genetic correlation, calculated using common SNPs, has been reported (.16) [19, 20].

It is also unknown whether the association between ASC and *psychotic experiences* is due to shared causes. Psychotic experiences display modest to moderate heritability across differing severity levels [21]. Likewise, subclinical autistic traits are highly heritable, again consistently across different severity levels [22]. If there is genetic overlap between clinical forms of these traits, one might hypothesise that the causes of autistic traits and psychotic experiences will also overlap. That is, one might predict that the genetic variants underlying autistic traits will also influence continuous variation in psychotic experiences in the general population.

The twin design can address this question, as it allows estimation of the extent of overlap in genetic and environmental influences between two traits. Prior studies of ASC and psychotic experiences have employed data from singletons [10–14], meaning that they were unable to test the degree of genetic and environmental overlap across these phenotypes. We therefore aimed to investigate the degree to which the causes of autistic traits across an 8-year period of childhood would account for *adolescent* psychotic experiences. Psychotic experiences comprise multiple factors [8, 23]; hence, we focused on *individual* psychotic experiences in adolescence. We also examined whether individuals with ASC displayed more psychotic experiences than typically developing individuals.

We expected genetic influences on autistic traits to account for a significant degree of variance in adolescent psychotic experiences, specifically negative symptoms [13, 14]. Individuals with ASC were expected to display more negative symptoms, in particular, than typically developing individuals.

## Methods

### Participants

Participants were taking part in the Twins Early Development Study (TEDS), a community sample of twins born in England and Wales between 1994 and 1996 [24]. When twins were aged 16, 10,874 families were invited to take part in the Longitudinal Experiences And Perceptions (LEAP) study, a study of psychotic experiences in the general population. In total, 5128 parents returned LEAP data, while 5074 twin pairs both returned questionnaires. Participating and nonparticipating families in LEAP were similar on various demographic characteristics (shown in Additional file 1). Autistic traits were assessed four times: at ages 8, 12, 14, and 16. All participants in LEAP who had autistic trait data at any prior age were included in the study. Exclusions were conducted for genetic syndromes, chromosomal abnormalities, perinatal/postnatal complications, and missing first contact or zygosity data. The final sample sizes are shown in Table 1. Zygosity was determined through parent report of twin resemblance and DNA testing [25]. TEDS has full ethical approval from the King’s College London, Institute of Psychiatry Ethics Committee. All participants provided informed consent before completing questionnaires.

**Table 1:** Interpretation of twin correlations

Component	Cross-twin correlation	Cross-trait cross-twin correlation
Additive genetic (A)	MZ correlation is higher than the DZ correlation	MZ correlation is higher than the DZ correlation
Shared environmental (C)	DZ correlation is greater than half the MZ correlation	DZ correlation is greater than half the MZ correlation
Nonshared environmental (E)	MZ correlation is lower than 1	MZ correlation is lower than the phenotypic correlation

Cross-twin correlation: correlation of one twin’s score on a trait with their co-twin’s score on the same trait; cross-trait cross-twin correlation: correlation of one twin’s score on a trait with their co-twin’s score on another

*MZ* monozygotic twins, *DZ* dizygotic twins

### Measures

#### Autistic traits

Autistic traits were assessed at all four ages. At ages 8 and 12 years, parents completed the Childhood Autism Spectrum Test (CAST [26]), a 31-item questionnaire (30 items were used at age 12 years due to the removal of an age-inappropriate item). Each question was answered ‘yes’ or ‘no’; possible ranges of scores are shown in Table 1. The measure was reliable (see Table 1) and stable between ages 8 and 12 years (*r* = .55, *p* < .001). Scores over 15 have 100 % sensitivity and 97 % specificity in detecting individuals at risk of ASC, with a positive predictive value of 50 % [27]. The CAST was divided into three previously published subscales [28]: social difficulties, communication atypicalities, and repetitive behaviours and interests.

The CAST is a measure for elementary school-aged children [26]; hence, parents completed the shortened Autism Spectrum Quotient (AQ [29, 30]) at ages 14 and 16 years. The AQ comprised 38 items at age 14 years and 28 items at age 16 years. Parents rated their agreement with each item on a 4-point scale. The AQ was reliable (see Table 1) and stable between ages 14 and 16 years (*r* = .67, *p* < .001). Individuals with ASC scored significantly higher on the AQ at ages 14 (*t*_2546_ = −9.43, *p* < .001) and 16 years (*t*_4894_ = −15.00, *p* < .001). The AQ was divided into subscales [29]: social difficulties, communication, imagination, attention to detail, and attention switching. Communication items were omitted from the scale at age 16 owing to space constraints.

#### ASC diagnoses

Parents of twins scoring over 15 on the CAST were invited to complete the Diagnostic and Well-Being Assessment (DAWBA [31]), a structured interview for establishing psychiatric diagnoses. Thirty-eight ASC-relevant items were administered over the telephone. The DAWBA effectively distinguishes between ASC cases and controls^30^, and has good inter-rater reliability (*κ* = 0.83) and internal consistency (*α* = 0.92) in this sample [32]. If either twin in a pair met DAWBA ASC criteria, families were invited to take part in the Social Relationships Study [33]. Two trained researchers visited the families to complete the Autism Diagnostic Observation Schedule [34] with the twins and Autism Diagnostic Interview-Revised [35] with the parents; these semi-structured assessments are considered the ‘gold standard’ in diagnosing ASC in research. In total, 32 participants with a confirmed diagnosis had Specific Psychotic Experiences Questionnaire (SPEQ) data available (29 males, 3 females).

#### Psychotic experiences

Psychotic experiences at age 16 were assessed using the SPEQ [8]. The SPEQ was constructed from six existing measures of psychotic experiences in adults. The wording of these measures was adapted for use with adolescents, and the age appropriateness of the items was established in terms of expert clinical opinion. Positive and cognitive psychotic experiences were assessed using four self-report measures: paranoia (15 items), hallucinations (9 items), cognitive disorganisation (11 items), and grandiosity (8 items). Negative symptoms were assessed via a self-report measure of anhedonia (10 items) and a parent measure of negative symptoms (9 items). The use of both a parent- and self-report measure of negative symptoms was designed to reflect recent recommendations that assessment of negative symptoms should include multiple informants [36]. One item (‘Has few or no friends’) was removed from negative symptoms owing to overlap with the autistic trait measures. Table 1 shows ranges of scores and internal consistencies. All subscales were stable across a 9-month period (*r* = .65–.74). Principal-components analysis supported the division of SPEQ into six subscales [8]. The measure has been validated against a similar measure, the Psychosis-Like Symptoms (PLIKS) scale [8, 37]. Individuals reporting having ‘definitely’ had psychotic experiences on the PLIKS scored significantly higher on all SPEQ subscales than those who did not (all *p* < .001). Individuals with relatives with schizophrenia or bipolar disorder (*N* = 420, 227 males, 193 females) scored significantly higher on all SPEQ subscales (*p* < .05), except for anhedonia and hallucinations (which showed a nonsignificant trend).

### Data analyses

Skewed measures were log transformed (Table 1). Sex and age effects can inflate twin correlations (described below). As a result, all scores were regressed for sex and age, as is standard behavioural genetic procedure [38]. Two sets of analyses were performed. Multivariate twin models were fitted to data on continuous autistic traits and psychotic experiences in the whole sample. Mean group differences in continuous SPEQ scores were then compared in individuals with a diagnosis of ASC and those without such a diagnosis.

Analyses were performed in the OpenMx [39] package of R [40]. OpenMx uses full information maximum likelihood when fitting models, meaning that missing data can be accounted for. Rather than excluding participants with any missing data, full information maximum likelihood includes participants with data for at least one age, meaning that the sample is not limited by the presence of missing data [39].

#### Phenotypic analyses

Phenotypic correlations (*r*_ph_) between measures were estimated from twin models.

Mean standardised SPEQ scores were compared across individuals with ASC and controls using independent *t* tests. In addition, we tested whether individuals with a family history of a psychotic disorder would display more autistic traits than those without such a family history, again using independent *t* tests. Welch’s degrees of freedom for unequal sample sizes were applied.

#### Twin analyses

The twin design seeks to partition variance in a phenotype into three components: additive genetic influences (A); shared environmental influences (C), which are common to both twins in a pair and cause similarity between them; and nonshared environmental influences (E), which are unique to each twin and create differences between them. These parameters are estimated on the basis of the phenotypic resemblance of monozygotic (MZ) twins, who share all their segregating DNA code, and dizygotic (DZ) twins, who share on average 50 % of their segregating DNA code.

Cross-twin correlations were used to establish the phenotypic similarity of MZ and DZ twins. These correlations, estimated from a saturated model of the observed data, were obtained separately for MZ and DZ twins, and involved correlating one twin’s score on a trait (e.g. autistic traits) with their co-twin’s score on the same trait. One can then gain an indication of the extent of A, C, and E influences by examining the MZ and DZ cross-twin correlations. Table 1 shows how the pattern of cross-twin correlations can be interpreted.

The multivariate twin design allows one to investigate the covariance between multiple traits by dividing the correlation between them into A, C, and E components. Cross-trait cross-twin correlations were estimated as a starting point and involved correlating one twin’s autistic traits with their co-twin’s psychotic experiences score. Again derived separately for MZ and DZ twins, these correlations can be used to obtain an initial indication of the extent to which A, C, and E influence the covariance between two traits. Table 1 shows how cross-trait cross-twin correlations can be interpreted.

**Fig. 1:**
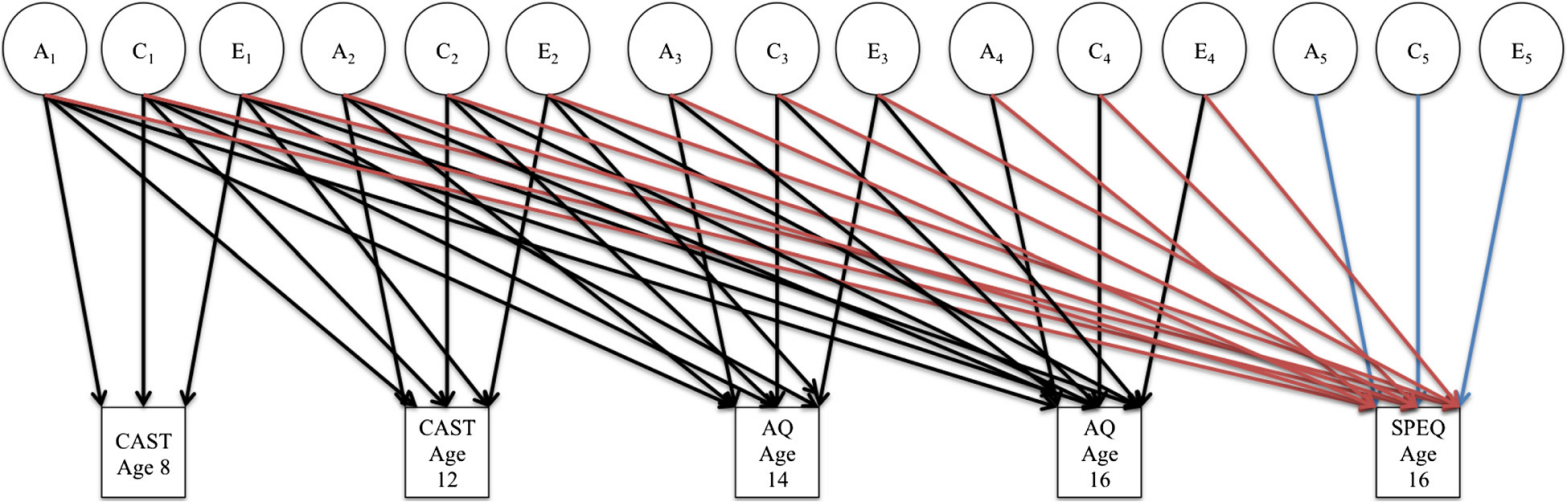
Path diagram of the Cholesky decomposition. Variables enclosed in *circles* are latent variables. The *red arrows* connecting A_1_–A_4_, C_1_–C_4_, and E_1_–E_4_ to SPEQ age 16 represent the sources of shared variance between autistic traits and SPEQ subscale scores. The *blue arrows*, which connect A_5_, C_5_, and E_5_ to SPEQ age 16, represent the residual variance in SPEQ subscales scores. The *black arrows* represent the paths between autistic traits at each age. *CAST* Childhood Autism Spectrum Test, *AQ* Autism Spectrum Quotient, *SPEQ* Specific Psychotic Experiences Questionnaire, *A* additive genetic influences, *C* shared environmental influences, *E* nonshared environmental influences

Structural equation twin-model fitting formally estimated A, C, and E. Cholesky decompositions were fitted to measures displaying sufficient covariance with one another (*r*_ph_ > .20 at age 16). These decompositions estimated the degree to which the causes of autistic traits could account for psychotic experiences. In Fig. 1, the pathways from latent variables (enclosed in circles) A_1_, C_1_, E_1_, A_2_, C_2_, E_2_, A_3_, C_3_, E_3_, A_4_, C_4_, and E_4_ to SPEQ subscales at 16 years denote the extent to which the causes of autistic traits influence psychotic experiences. In squaring these estimates, one can derive the proportions of variance explained. A_5_, C_5_, and E_5_ represent residual variance in SPEQ scores that is unique to psychotic experiences.

In the first instance, all estimates were free to differ by sex (*quantitative sex limitation*), and were then equated across sexes to test the significance of any sex differences. Parameters were then successively dropped from the ACE Cholesky decomposition by fixing them to 0. The fit of the Cholesky decompositions was compared against a baseline-saturated model of observed means and variances. The fit of each model was summarised by the fit statistic which was −2 times the log likelihood of the data (−2LL); differences in −2LL between two models are *χ*^2^ distributed, with degrees of freedom equivalent to the difference in number of parameters. Significant *χ*^2^ results suggest a model is a significantly poorer fit than the saturated model. Model fit was further assessed using Akaike’s Information Criteria (AIC) and Bayesian Information Criteria (BIC). Best-fitting models were selected based on the most negative BIC value.

Effects of two potential confounders were then tested: general cognitive ability and internalising traits. A composite g-score was computed when twins were aged 7, 12, 14, and 16 years [41]. At age 7, twins completed three measures from the Wechsler Intelligence Test for Children (WISC [42]): picture completion, vocabulary, and similarities. Twins also completed the McCarthy Conceptual Groups test [43]. At age 12, twins completed the WISC vocabulary, picture completion, and general knowledge tests [42] as well as the Raven’s Standard Progressive Matrices [44]. At ages 14 and 16, the Raven’s Standard Progressive Matrices and WISC vocabulary tests were administered. At each age, scores on all completed measures were standardised; g-score was calculated as the average of the standardised test scores at each age.

Internalising traits were a second potential confounder. They were assessed using two self-report measures, the Childhood Anxiety Sensitivity Index [45] and Short Moods and Feelings Questionnaire [46]. Analyses were repeated with each of these confounders regressed out of all measures.

## Results

### Phenotypic analyses

Descriptive statistics are in Table 2 and are shown by sex and zygosity in Additional file 2. Table 2 shows *r*_ph_ estimates; at age 16 years, the highest estimates were between autistic traits and negative symptoms (*r*_ph_ = .47, *p* < .01), and autistic traits and anhedonia (*r*_ph_ = .30, *p* < .01). r_ph_ showed a trend for increasing with age; *r*_ph_ between autistic traits and negative symptoms increased from .27 at age 8 years to .47 at age 16 years, and from .14 to .30 for anhedonia. All other estimates were modest (.00–.21); therefore, only negative symptoms and anhedonia were included in twin-model fitting.

**Table 2:** Sample size, descriptive statistics, and phenotypic correlations

Sample sizes						
Age	*N* families contacted	MZM	DZM	MZF	DZF	DZOS
8 years	13,941	858	804	1046	874	1659
12 years	8438	814	775	1050	895	1601
14 years	11,118	529	463	662	548	911
16 years	10,874	738	686	1043	905	1560
Measure descriptive statistics						
Measure	Cronbach’s *α*	Possible range of scores		$\overline{x}$ (SD)		Skew
CAST age 8	0.71	0–31		4.85 (3.09)		0.99 (−0.64)
CAST age 12	0.73	0–30		4.62 (3.15)		1.24 (−0.48)
AQ age 14	0.80	0–87		36.78 (12.16)		0.24 (0.21)
AQ age 16	0.80	0–80		24.04 (10.62)		0.39 (0.36)
SPEQ paranoia	0.93	0–72		12.17 (10.62)		1.56 (−0.61)
SPEQ hallucinations	0.88	0–45		4.66 (6.01)		2.08 (0.22)
SPEQ cognitive disorganisation	0.77	0–11		3.96 (2.85)		0.44 (−0.63)
SPEQ grandiosity	0.86	0–24		5.32 (4.43)		1.19 (−0.41)
SPEQ anhedonia	0.78	0–50		16.36 (7.93)		1.14 (0.17)
SPEQ negative symptoms	0.86	0–27		2.63 (3.63)		2.32 (0.45)
Phenotypic correlations						
	Autistic traits age 8	Autistic traits age 12		Autistic traits age 14		Autistic traits age 16
Paranoia	.02 (ns)	.06*		.07**		.10**
Hallucinations	.07**	.10**		.13**		.14**
Cognitive disorganisation	.08**	.12**		.17**		.21**
Grandiosity	.04*	.08**		.04 (ns)		.00 (ns)
Anhedonia	.14**	.18**		.24**		.30**
Negative symptoms	.27**	.32**		.40**		.47**

All means are given for untransformed scores; skew values are shown for the untransformed measures, and then the standardised residuals used in analyses in parentheses. All Ns are number of twin pairs with data available at each age

*SPEQ* Specific Psychotic Experiences Questionnaire, *CAST* Childhood Autism Spectrum Test, *AQ* Autism Spectrum Quotient, *ns* nonsignificant

*p < .05; **p < .01

Cross-sectionally, negative symptoms showed stronger associations with attention switching (*r*_ph_ = .45, *p* < .01) and social difficulties (*r*_ph_ = .44, *p* < .01) than the other AQ subscales, as did anhedonia (*r*_ph_ = .25–.37). A full table of *r*_ph_ between psychotic experiences and the autistic trait subscales is shown in Additional file 3.

### Twin analyses

#### Twin correlations

Twin correlations are shown in Table 3. For autistic traits, MZ cross-twin correlations were higher than DZ estimates, suggesting A. MZ estimates were less than 1, suggesting E, while DZ estimates all exceeded half the MZ estimates, implicating C. For anhedonia and negative symptoms, MZ cross-twin correlations were higher than DZ statistics, implicating A. MZ estimates were all below 1, suggesting E. In all instances, the MZ cross-trait cross-twin correlations between negative symptoms and autistic traits, and anhedonia and autistic traits were higher than the DZ estimates. This suggests that genetic factors played a role in the covariance between autistic traits and negative symptoms, and autistic traits and anhedonia.

**Table 3:** Twin correlations

Cross-twin correlations										
	MZM		DZM		MZF		DZF		DZOS	
	Estimate	95 % CI	Estimate	95 % CI	Estimate	95 % CI	Estimate	95 % CI	Estimate	95 % CI
Autistic traits age 8	.83	.81/.85	.37	.35/.39	.79	.76/.82	.45	.40/.49	.48	.44/.49
Autistic traits age 12	.79	.77/.82	.32	.29/.35	.76	.73/.79	.51	.47/.55	.41	.38/.45
Autistic traits age 14	.91	.88/.93	.54	.51/.56	.88	.85/.89	.62	.60/.66	.51	.47/.55
Autistic traits age 16	.92	.90/.95	.51	.48/.55	.82	.80/.87	.58	.55/.61	.46	.44/.50
Anhedonia	.37	.31/.39	.19	.18/.22	.45	.42/.46	.17	.13/.19	.14	.12/.15
Negative symptoms	.80	.78/.82	.59	.55/.60	.81	.78/.82	.52	.47/.54	.47	.44/.48
Cross-trait cross-twin correlations										
	MZM		DZM		MZF		DZF		DZOS	
	Estimate	95 % CI	Estimate	95 % CI	Estimate	95 % CI	Estimate	95 % CI	Estimate	95 % CI
Autistic traits age 8—anhedonia	.18	.15/.22	.07	.03/.10	.22	.19/.23	.03	.01/.05	.08	.05/.09
Autistic traits age 12—anhedonia	.20	.17/.23	.00	−.05/.04	.23	.19/.26	.07	.03/.09	.11	.05/.13
Autistic traits age 14—anhedonia	.24	.15/.27	.16	.09/.20	.32	.21/.34	.12	.08/.15	.14	.04/.17
Autistic traits age 16—anhedonia	.28	.24/.29	.15	.09/.17	.31	.29/.33	.11	.06/.12	.11	.09/.13
Autistic traits age 8—negative symptoms	.22	.19/.23	.18	.15/.19	.24	.21/.25	.19	.15/.22	.20	.18/.22
Autistic traits age 12—negative symptoms	.34	.31/.35	.24	.22/.25	.29	.27/.31	.19	.17/.22	.19	.15/.22
Autistic traits age 14—negative symptoms	.37	.31/.44	.30	.22/.36	.32	.23/.36	.25	.18/.29	.24	.21/.27
Autistic traits age 16—negative symptoms	.43	.42/.45	.31	.29/.34	.36	.34/.38	.30	.28/.36	.21	.19/.23

*MZM* monozygotic male, *DZM* dizygotic male, *MZF* monozygotic female, *DZF* dizygotic female, *DZOS* dizygotic opposite sex

### Twin-model fitting

Five-variable Cholesky decompositions were fitted to (1) autistic traits and anhedonia, and (2) autistic traits and negative symptoms. Model-fit statistics and parameter estimates are shown in Table 4.

**Table 4:** Twin-model fit statistics and parameter estimates

								Comparative fit with saturated model				Comparative fit with full ACE Cholesky			
Model			−2LL	df		Par.	BIC	Δ*χ* ^2^	Δdf	*p*	AIC	Δ*χ* ^2^	Δdf	*p*	AIC
Autistic traits and anhedonia (five-variable decompositions)															
Saturated			63,287.21	27,375		260	−158948.70	–	–	–	–	–	–	–	–
ACE sex diff.			63,489.32	27,535		100	−160045.50	202.11	160	<.05	−117.89	–	–	–	–
ACE const. ^a^			63,619.71	27,580		55	−160280.40	332.50	205	<.001	−77.50	130.39	45	<.001	40.39
AE const.			63,784.42	27,595		40	−160237.50	497.20	220	<.001	57.20	164.71	15	<.001	134.71
CE const.			64,974.16	27,595		40	−159047.80	1686.94	220	<.001	1246.94	1345.45	15	<.001	1324.45
E const.			71,621.98	27,610		25	−152521.80	8334.77	235	<.001	7864.77	8002.27	30	<.001	7942.27
Autistic traits and parent-rated negative symptoms (five-variable decompositions)															
Saturated			60,014.32	27,393		260	−162367.70	–	–	–	–	–	–	–	–
ACE sex diff.			60,189.54	27,553		100	−163491.40	175.22	160	.19	−144.78	–	–	–	–
ACE const. ^a^			60,319.68	27,598		55	−163726.60	305.35	205	<.001	−104.65	130.13	45	<.001	40.13
AE			60,542.65	27,613		40	−163625.40	528.33	220	<.001	−7.02	222.98	15	<.001	192.98
CE			61,994.75	27,613		40	−162173.31	1980.42	220	<.001	1540.42	1675.07	15	<.001	1645.07
E			70,414.98	27,628		25	−153874.80	10400.66	235	<.001	9930.66	10095.31	30	<.001	10035.31
Parameter estimates from best-fitting decompositions															
	Autistic traits age 8			Autistic traits age 12			Autistic traits age 14			Autistic traits age 16			Variance specific to psychotic experiences		
	A_1_	C_1_	E_1_	A_2_	C_2_	E_2_	A_3_	C_3_	E_3_	A_4_	C_4_	E_4_	A_5_	C_5_	E_5_
Anhedonia	.02 (.00/.05)	.01 (.00/.05)	.00 (−.01/.01)	.00 (.00/.03)	.01 (.00/.04)	.00 (−.01/.01)	.02 (.00/.05)	.01 (.00/.04)	.00 (.00/.01)	.01 (.00/.05)	.00 (−.01/.03)	.02 (.00/.03)	.32 (.26/.36)	.00 (−.05/.06)	.58 (.55/.61)
Total	.03			.01			.03			.03			.90		
Neg sym	.02 (.00/.04)	.10 (.04/.22)	.00 (.00/.01)	.01 (.00/.03)	.06 (.01/.17)	.00 (.00/.01)	.05 (.03/.10)	.00 (−.01/.01)	.01 (.00/.01)	.04 (.01/.07)	.00 (−.02/.02)	.02 (.01/.03)	.45 (.40/.50)	.10 (−.18/.18)	.14 (.13/.15)
Total	.12			.07			.06			.06			.69		

AE, CE, and E models are submodels within the ACE model that fit best. A_1_–A_4_, C_1_–C_4_, and E_1_–E_4_ are the paths shown in red in Fig. 1, and represent the influence of genetic and environmental causes of autistic traits on psychotic experiences, A_5_, C_5_, and E_5_ are residual pathways (shown in blue in Fig. 1), which represent the genetic and environmental influences on psychotic experiences that are independent of autistic traits

*Saturated* saturated model of means and covariance, *ACE sex diff.* full ACE Cholesky decomposition, which included quantitative sex differences for all parameters, *ACE const.* constrained ACE Cholesky decomposition, with parameters fixed to be equal across sexes, *−2LL* fit statistic, *df* degrees of freedom, *Par.* parameters, *AIC* Akaike’s Information Criteria, *BIC* Bayesian Information Criteria, *A* additive genetic influences, *C* shared environmental influences, *E* nonshared environmental influences

^a^Indicates best-fitting model

#### Autistic traits and anhedonia

An ACE decomposition with no sex differences best fit autistic traits and anhedonia. Paths A_1_–A_4_, C_1_–C_4_, and E_1_–E_4_ in Fig. 1 denote the genetic and environmental influences on childhood autistic traits that also influence anhedonia at age 16 years. Summing these paths gives the total proportion of variance in anhedonia explained by autistic traits. In total, 10 % of the variance in anhedonia was explained by autistic traits. Of this variance, half (5 %) was explained by A influences on autistic traits, 3 % was due to C influences shared with autistic traits, and 2 % was due to E. Of the 90 % of variance in anhedonia that was independent of autistic traits, 32 % was explained by A and 58 % by E. Anhedonia was 37 % heritable, yet only 5/37 = 13.5 % of this heritability was due to genetic influences shared with childhood autistic traits.

#### Autistic traits and negative symptoms

An ACE Cholesky composition with no sex differences best fit autistic traits and negative symptoms. A, C, and E influences on autistic traits between ages 8–16 years (A_1_–A_4_, C_1_–C_4_, and E_1_–E_4_) accounted for 31 % of the variance in negative symptoms. Of this shared variance, 12 % was explained by A influences on autistic traits, 16 % was due to C, and 3 % was due to E. Of the 69 % variance in negative symptoms that was independent of autistic traits, most (45 %) was explained by A, while C and E comprised 10 % and 14 %, respectively. Negative symptoms were 57 % heritable, yet only 12/57 = 21 % of this heritability was due to genetic influences shared with childhood autistic traits.

#### Controlling for confounders

Controlling for g-score or internalising traits resulted in nonsignificant reductions in the phenotypic correlations. Consequently, we did not repeat twin-model fitting on these adjusted scores (contact the first author for details).

### Individuals with ASC

**Fig. 2:**
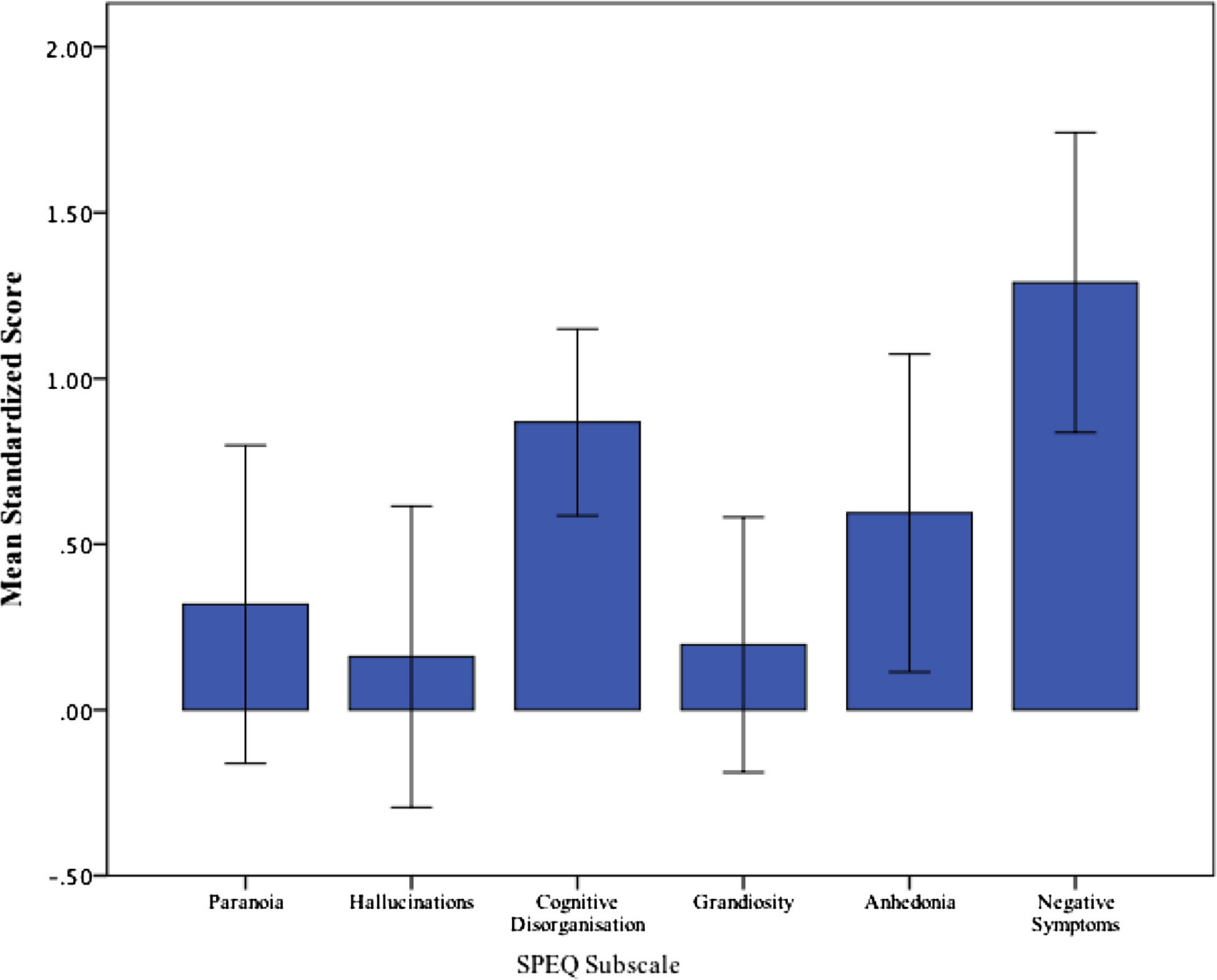
Mean standardised scores on the SPEQ subscales for participants with ASC. *Error bars* represent 95 % confidence intervals; all means are given as mean standardised residual scores. *SPEQ* Specific Psychotic Experiences Questionnaire

Mean standardised SPEQ scores for participants with ASC are shown in Fig. 2. Mean scores for controls were 0 owing to the standardisation of scores. Individuals with ASC displayed significantly higher scores than controls for cognitive disorganisation (*t*_24.54_ = −6.68, *p* < .001, *r* = .80), anhedonia (*t*_24.20_ = −2.55, *p* < .05, *r* = .46), and negative symptoms (*t*_24.20_ = −2.55, *p* < .05, *r* = .80).

### Family history of psychotic disorders

Mean AQ scores at age 16 were virtually identical across individuals with a first-degree relative with a psychotic disorder ($\overline{x}$ = 24.76 [SD = 11.68]) and those without ($\overline{x}$ = 24.14 [SD = 10.81]). Mean scores did not differ significantly across individuals with and without a family history of a psychotic disorder, *t*_489.53_ = −1.04, *p* = .30.

## Discussion

Childhood autistic traits, and diagnosed ASC, were associated with negative psychotic experiences in adolescence, but not positive or cognitive psychotic experiences. As expected, twin modelling revealed a degree of shared genetic and environmental influences between these traits, yet this was modest. The majority of variance in adolescent negative symptoms was independent of autistic traits. In addition, there was no indication of an elevation in autistic traits for individuals with a family history of psychotic disorders. Thus, our results imply a limited association between autistic traits and psychotic experiences. These findings could not be accounted for by general cognitive ability or internalising traits.

Our findings concur with two previous studies in showing that autistic traits are linked with negative symptoms, in particular [13, 14], yet associations with positive and cognitive psychotic experiences were weaker. These findings seemingly contradict two community-based studies [10, 11]. One possibility is that the use of different raters for autistic traits and these psychotic experiences in our study dampened the associations between them. This, however, is unable to explain why the association with negative symptoms held across both parent and self reports. It is notable that, while the association was weaker than with negative symptoms, cognitive psychotic experiences still showed a stronger correlation with autistic traits than positive symptoms in our study. It may, therefore, be that the previous findings were being driven by cognitive symptoms.

One of the core questions posed in this study was whether or not autistic traits would share genetic causes with psychotic experiences. Could the genetic variants associated with both ASC and schizophrenia, such as calcium-signalling genes [19] and 16p11.2 duplications [47, 48], mean that genetic factors might also play a role in influencing the co-occurrence of autistic traits and psychotic experiences in the general population? The genetic influences on autistic traits accounted for some variance in negative symptoms; however, this was only a small proportion of variance. More importantly, autistic traits were not as strongly linked with positive symptoms, such as paranoia, emphasising the possibility that any genetic associations between autistic traits and psychotic experiences are constrained to negative psychotic experiences. As such, we do not predict that studies of the genetic basis of psychotic experiences are likely to prove informative when attempting to understand the aetiology of ASC.

In light of recent findings [19, 47, 48], some have argued that clinical ASC and schizophrenia are part of a continuum of neurodevelopmental causality [49]. Indeed, there is evidence of some genetic and environmental overlap across autistic traits and traits of other conditions, including ADHD and anxiety [50, 51]. The present findings can be taken as partial support to the idea of a continuum of neurodevelopmental conditions, with two key caveats. First, the relationship between autistic traits and psychotic experiences was limited to negative psychotic experiences. Second, the majority of the genetic and environmental influences on psychotic experiences were distinct from autistic traits, tallying with SNP-based evidence reporting low genetic correlations between ASC and schizophrenia [19]. As such, these findings converge to suggest that while there is some overlap between these phenotypes, they are largely separate, with low genetic overlap.

Given the modest role played by shared environment in autistic traits [22] and psychotic experiences [21], it is surprising that such influences on autistic traits accounted for some variance in psychotic experiences. The net influence of shared environment on anhedonia was weak, however. The use of the same rater for negative symptoms and autistic traits may also have inflated the role of shared environment in their covariance.

This study was not free from limitations. As with all studies of co-occurrence of different traits that rely on questionnaire data, there is some risk that items can be interpreted in different ways by different people and as such overlap could be for heterogeneous reasons. However, all the evidence here suggests that each of our measures is capturing what they aim to capture, and thus, any overlap is due to true co-occurrence of psychotic experiences and autistic traits in individuals.

A consideration is whether or not the association between autistic traits, anhedonia and negative symptoms, was driven by phenotypic similarity of the items. In the first instance, we removed an ostensibly overlapping item from the negative symptoms subscale of the SPEQ (‘Has few or no friends’). Additionally, the correlations between scores on these measures were not total, indicating a degree of independence between the measures. Finally, our measures of autistic traits and psychotic experiences both have good construct and content validity, as outlined in the Methods‘’ section.

Some may query the use of self-report measures in individuals with ASC, but it needs to be noted that the elevation in negative symptoms in individuals with ASC was evident not only for self-reported anhedonia, but also for parent-rated negative symptoms. It is worth noting, however, that autistic traits were rated by parents only. Future research should endeavour to obtain ratings of autistic traits from multiple informants, for example teachers in addition to parents. Finally, the use of a large sample, a requisite for twin-model fitting, necessitated the use of questionnaire measures over in-depth assessments. As such, the sample comprised not only individuals displaying very high scores on the measures, but also a considerable number of individuals displaying lower scores, resulting in skewed measures. This, however, is to be expected in large, community-based studies of psychopathology.

While we had longitudinal data on autistic traits, we did not have longitudinal data on psychotic experiences. Longitudinal cross-lagged twin models can test whether one phenotype directly leads to another e.g. [51], but the absence of data on psychotic experiences at multiple ages in our sample meant that this was not possible in our study. Nevertheless, this would represent an important future direction for research.

One might question whether these twin findings generalise to singletons. Neither autistic traits [52] nor psychotic experiences [8] appear elevated in twins relative to singletons. Studies of singletons have also reported that psychotic experiences are elevated in individuals with ASC [10–14].

## Conclusions

These findings demonstrate that autistic traits and ASC were linked with negative symptoms in adolescence, such as anhedonia and affective flattening, but less so with cognitive or positive psychotic experiences. In clinics, therefore, one might expect to see individuals with ASC present with more negative symptoms, rather than positive symptoms, such as hallucinations and paranoia. Despite the co-occurrence of autistic traits and ASC with negative symptoms, however, genetic overlap between them was low. There remains a considerable amount of work to be done to fully unravel the genetic basis of autistic traits and psychotic experiences. As we learn more about the causes of these phenotypes, on the basis of the present findings, we do not predict that the same genetic variants will be identified as associated with autistic traits and psychotic experiences.

## Additional files

Additional file 1:
**Characteristics of participating and nonparticipating families in LEAP. **Characteristics of participants who returned LEAP questionnaires compared with those who did not, including sex, zygosity, ethnicity, and maternal education level. Additional file 2:
**Descriptive statistics on all measures by sex and zygosity.** Mean scores and standard deviations on measures of autistic traits at all ages and the six psychotic experience subscales, including ANOVAs. Additional file 3:
**Phenotypic correlations between autistic trait subscales and SPEQ subscales.** Correlations between subscales of the autistic trait measures at all ages and the six psychotic experiences subscales.

## Abbreviations

A: additive genetic influences
AIC: Akaike’s Information Criteria
BIC: Bayesian Information Criteria
C: shared environmental influences
E: nonshared environmental influences
*r*_ph_: phenotypic correlation
