# Supplementary material for: A longitudinal twin study of the association between childhood autistic traits and psychotic experiences in adolescence
Source: Mol Autism. 2015 Jul 22;6:44. doi: 10.1186/s13229-015-0037-9 (PMC4509468; doi:10.1186/s13229-015-0037-9)
Supplement: Additional file 1: — Characteristics of participating and nonparticipating families in LEAP. Characteristics of participants who returned LEAP questionnaires compared with those who did not, including sex, zygosity, ethnicity, and maternal education level. [file 13229_2015_37_MOESM1_ESM.pdf]

Characteristics of participating and non-participating families in LEAP

	Participating in LEAP	Non-Participating in LEAP
% Male	45%	53%
% Monozygotic	35%	32%
% White	94%	91%
% Mothers with one or more A-levels	16%	12%

LEAP: Longitudinal Experiences And Perceptions project; A-levels are advanced examinations taken at age 17-18 in England and Wales
