# Supplementary material for: A longitudinal twin study of the association between childhood autistic traits and psychotic experiences in adolescence
Source: Mol Autism. 2015 Jul 22;6:44. doi: 10.1186/s13229-015-0037-9 (PMC4509468; doi:10.1186/s13229-015-0037-9)
Supplement: Additional file 2: — Descriptive statistics on all measures by sex and zygosity. Mean scores and standard deviations on measures of autistic traits at all ages and the six psychotic experience subscales, including ANOVAs. [file 13229_2015_37_MOESM2_ESM.pdf]

Descriptive statistics on all measures by sex and zygosity for a) autistic traits and b) psychotic experiences

Zygosity	CAST Age 8 (SD) [a]	CAST Age 12 (SD) [b]	AQ Age 14 (SD) [c]	AQ Age 16 (SD) [d]
**Autistic Traits**				
MZ Male	5.35 (3.22)	4.96 (3.23)	36.95 (12.16)	25.04 (10.34)
DZ Male	5.33 (3.15)	4.98 (3.21)	38.45 (12.50)	25.42 (10.77)
MZ Female	4.20 (2.68)	3.96 (2.84)	35.43 (11.57)	22.73 (10.31)
DZ Female	4.55 (2.98)	4.29 (2.94)	36.23 (11.54)	22.80 (9.86)
DZ Opposite-Sex	5.04 (3.24)	4.93 (3.32)	37.28 (12.72)	24.57 (11.15)

**Psychotic Experiences**						
Zygosity	Paranoia (SD) [e]	Hallucinations (SD) [f]	Cognitive Disorganization (SD) [g]	Grandiosity (SD) [h]	Anhedonia (SD) [i]	Negative Symptoms (SD) [j]
MZ Male	11.11 (9.88)	4.09 (5.80)	3.22 (2.69)	5.79 (4.47)	31.52 (8.03)	2.60 (3.29)
DZ Male	12.04 (10.40)	4.31 (5.82)	3.57 (2.82)	5.86 (4.61)	31.38 (7.88)	2.99 (4.01)
MZ Female	12.30 (10.83)	4.72 (5.95)	4.30 (2.82)	4.90 (4.24)	35.56 (7.52)	2.36 (3.36)
DZ Female	12.06 (10.21)	4.85 (6.28)	4.55 (2.88)	4.73 (4.18)	35.23 (7.23)	2.44 (3.62)
DZ Opposite-Sex	12.69 (11.13)	4.93 (6.04)	3.91 (2.83)	5.50 (4.54)	33.47 (8.05)	2.78 (3.77)

CAST: Childhood Autism Spectrum Test; AQ: Autism Spectrum Quotient

[a] *Significant main effect of sex,* $F_{1,3866}=147.21$, $p<.001$

[b] *Significant main effect of sex,* $F_{1,3994}=127.52$, $p<.001$, *and zygosity,* $F_{1,3994}=6.67$, $p<.05$

[c] *Significant main effect of sex,* $F_{12544}=33.32$, $p<.001$, *and zygosity,* $F_{1,2544}=4.78$, $p<.05$; *significant interaction,* $F_{1,2544}=5.25$, $p<.05$

[d] *Significant main effect of sex,* $F_{1,4892}=118.02$, $p<.001$; *significant interaction between sex and zygosity,* $F_{1,4892}=7.38$, $p<.01$

[e] *Significant main effect of sex,* $F_{1,4868}=6.61$, $p<.05$, *and zygosity,* $F_{1,4868}=4.20$, $p<.05$

[f] *Significant main effect of sex,* $F_{1,4876}=15.27$, $p<.001$, *and zygosity,* $F_{1,4876}=4.29$, $p<.05$

[g] *Significant main effect of sex,* $F_{1,4870}=143.70$, $p<.001$, *and zygosity,* $F_{1,4870}=7.95$, $p<.01$

[h] *Significant main effect of sex,* $F_{1,4872}=49.87$, $p<.001$

[I] *Significant main effect of sex,* $F_{1,4830}=318.31$, $p<.001$

[j] *Significant main effect of sex,* $F_{1,4885}=27.63$, $p<.001$; *main effect of zygosity approached significance,* $F_{1,4885}=3.93$, $p=.05$; *significant interaction between sex and zygosity,* $F_{1,4885}=5.95$, $p<.05$
