# Supplementary material for: A longitudinal twin study of the association between childhood autistic traits and psychotic experiences in adolescence
Source: Mol Autism. 2015 Jul 22;6:44. doi: 10.1186/s13229-015-0037-9 (PMC4509468; doi:10.1186/s13229-015-0037-9)
Supplement: Additional file 3: — Phenotypic correlations between autistic trait subscales and SPEQ subscales. Correlations between subscales of the autistic trait measures at all ages and the six psychotic experiences subscales. [file 13229_2015_37_MOESM3_ESM.pdf]

Phenotypic correlations between autistic trait subscales and SPEQ subscales

	Paranoia	Hallucinations	Cognitive Disorganisation	Grandiosity	Anhedonia	Negative Symptoms
**CAST subscales at age 8**						
Social difficulties	.03 (ns)	.03 *	.03 (ns)	.06 **	.13 **	.23 **
Communication atypicalities	.06 **	.04 **	.10 **	.04 *	.16 **	.31 **
Repetitive behaviors and interests	.06 **	.05 **	.04 *	.08 **	.09 **	.16 **
**CAST subscales at age 12**						
Social difficulties	.02 (ns)	.02 (ns)	.03 (ns)	.03 *	.15 **	.28 **
Communication atypicalities	.09 **	.07 **	.15 **	.07 **	.17 **	.36 **
Repetitive behaviors and interests	.09 **	.07 **	.07 **	.13 **	.12 **	.18 **
**AQ subscales at age 14**						
Communication atypicalities	.12 **	.10 **	.17 **	.05 **	.20 **	.39 **
Social difficulties	.07 **	.08 **	.09 **	.01 (ns)	.27 **	.35 **
Imagination	-.01 (ns)	.02 (ns)	.07 **	.03 (ns)	.19 **	.27 **
Attention switching	.14 **	.10 **	.17 **	.06 **	.22 **	.34 **
Attention to detail	.07 **	.07 **	.00 (ns)	.07 **	.05 *	.00 (ns)
**AQ subscales at age 16**						
Social difficulties	.12 **	.09 **	.14 **	-.02 (ns)	.37 **	.44 **
Imagination	.00 (ns)	.01 (ns)	.12 **	.00 (ns)	.17 **	.33 **
Attention switching	.14 **	.11 **	.22 **	.03 (ns)	.25 **	.45 **
Attention to detail	.04 *	.08 **	.00 (ns)	.10 **	.07 **	.04 *

*** p<.001; * p<.05; ns: non-significant*

CAST: Childhood Autism Spectrum Test; AQ: Autism Spectrum Quotient
